# Prevalence of metabolic syndrome and its risk factors in Kerala, South India: Analysis of a community based cross-sectional study

**DOI:** 10.1371/journal.pone.0192372

**Published:** 2018-03-27

**Authors:** S. Harikrishnan, Smitha Sarma, G. Sanjay, P. Jeemon, M. N. Krishnan, K. Venugopal, P. P. Mohanan, L. Jeyaseelan, K. R. Thankappan, G. Zachariah

**Affiliations:** 1 Department of Cardiology, Sree Chitra Tirunal Institute for Medical Sciences and Technology, Thiruvananthapuram, Kerala, India; 2 Department of Preventive Medicine, Feinberg School of Medicine, Northwestern University, Chicago, Illinois, United States of America; 3 Achutha Menon Centre for Health Science Studies, Sree Chitra Tirunal Institute for Medical Sciences and Technology, Thiruvananthapuram, Kerala, India; 4 Department of Cardiology, Government Medical College, Kozhikode, Kerala, India; 5 Department of Cardiology, Pushpagiri Hospital, Tiruvalla, Kottayam, Kerala, India; 6 Department of Cardiology, Westfort High-tech Hospital, Thrissur, Kerala, India; 7 Department of Biostatistics, Christian Medical College, Vellore, Tamil Nadu, India; 8 Department of Cardiology, Mother Hospital, Thrissur, Kerala, India; East Tennessee State University, UNITED STATES

## Abstract

**Background:**

Coronary Artery Disease (CAD) is a leading cause of death and disability in Kerala, India. Metabolic syndrome (MS) is a constellation of established risk factors for CAD. We aimed to estimate the prevalence of MS and evaluate the association between MS and CAD using a community-based sample population.

**Methods:**

A cross-sectional community based survey was conducted in urban and rural areas of Kerala in 2011. We included 5063 individuals for analysis. Age standardized prevalence of MS, associated diagnoses (hypertension, diabetes and hypercholesterolemia) and other potential risk factors were assessed for men and women in both urban and rural locations. Univariate and multivariate logistic regression models were developed to identify participant characteristics that are associated with MS.

**Results:**

After standardization for age and adjustment for sex and urban-rural distribution, the prevalence of metabolic syndrome in Kerala was 24%, 29% and 33% for the NCEP ATP III, IDF and AHA/NHLBI Harmonization definitions, respectively. The mean (SD) age of the participants was 51 (14) years, and 60% were women. Women had a higher prevalence of MS than men (28% versus 20% for ATP III, p<0.001). Similarly, participants living in urban areas had higher prevalence of MS than their rural counterparts (26% versus 22%, p<0.001). Elevated body mass index, older age, and female sex were associated with MS in an adjusted multivariate model. The propensity for definite CAD was 1.7 times higher in individuals with MS defined based on ATP III criteria compared to those without MS (Adjusted OR = 1.69; 95% CI: 1.3–2.2, p<0.001).

**Conclusions:**

One of four to one of three adult individuals in Kerala have MS based on different criteria. Higher propensity for CAD in individuals with MS in Kerala calls for urgent steps to prevent and control the burden of metabolic conditions.

## Introduction

Metabolic syndrome (MS) is an inter-related cluster of metabolic abnormalities involving glucose and lipid dysregulation, abdominal obesity and elevated blood pressure [1]. It is a premorbid condition that develops in the setting of insulin resistance and factors such as poor diet, physical inactivity, obesity, and genetics play a contributing role. Metabolic syndrome increases the risk for development of type 2 diabetes mellitus, coronary artery disease (CAD), and other cardiovascular diseases and has been shown to independently increase all-cause mortality [2,3]. Widely accepted definitions for the diagnosis of MS (Table 1) include criteria developed by the National Cholesterol Education Program’s Adult Treatment Panel III (ATP III), the International Diabetes Federation (IDF), and the American Heart Association/National Heart Lung and Blood Institute (Harmonization) [4–6]. In addition to serving as a predictive tool for the development of cardiovascular disease and type 2 diabetes, MS identification allows for the development and evaluation of targeted lifestyle interventions to combat the rising burden of non-communicable diseases.

**Table 1:** Diagnostic criteria for metabolic syndrome for ATP III, IDF and Harmonization definitions.

	National Cholesterol Education Program’s Adult Treatment Panel III (ATP III)	International Diabetes Federation (IDF)	Harmonization criterion for Asian Indians (Harmonization)
**Abdominal obesity**			
**Men**	>102 cm	≥90 cm*	≥90 cm*
**Women**	>88 cm	≥80 cm*	≥80 cm*
**Hyperglycemia**^a^	≥100 mg/dl	≥100 mg/dL	≥100 mg/dL
**Hypertriglyceridemia**^b^	≥150 mg/dl	≥150 mg/dL	≥150 mg/dL
**Low HDL cholesterol**			
**Men**	< 40 mg/dL	< 40 mg/dL	< 40 mg/dL
**Women**	< 50 mg/dL	< 50 mg/dL	< 50 mg/dL
**Elevated blood pressure**^a^	≥130/> = 85 mmHg	≥130/≥85 mm Hg	≥130/≥85 mmHg
**Diagnostic criteria**	3/5 risk factors	Must have abdominal obesity + 2 other risk factors	3/5 risk factors

* South Asian waist circumference cut off used

^a^ Includes all patients who are diagnosed and treated for diabetes mellitus and hypertension, respectively

^b^ As per the definition, individuals on specific triglyceride lowering treatments must be counted. However, this study did not include such individuals as this information was not collected.

The state of Kerala has the best health indicators in India in terms of maternal and infant mortality but has experienced a rising burden of non-communicable diseases in recent decades [7]. Coronary artery disease is the leading cause of death in Kerala, accounting for 31% of mortality among men and 18% among women [8]. Moreover, CAD is on the rise: the prevalence of definite CAD was 3.5% in 2011, a three-fold increase from 1993 [9]. Kerala also has a high prevalence of hypertension (crude estimates range from 32% to 55%) and the highest prevalence of diabetes in the country (crude: 16% to 20%) [10–14]. Alarmingly, risk factors for these non-communicable diseases such as obesity, substance use and physical inactivity appear to be increasing in prevalence [15].

Here, we present the prevalence of MS in Kerala and its association with CAD. We believe that MS, as a composite of several risk factors, is a useful metric to gauge the cardiovascular health of the population of Kerala. Additionally, when trended over time, estimates of MS prevalence will prove useful in evaluating the effectiveness of population-level lifestyle interventions. While previous studies including those by Vijayakumar et al (2009), Thankappan et al (2010), and Sathish et al (2012) have estimated prevalence of the various risk factors for non-communicable disease in Kerala including many of the components of MS, ours is the first contemporary published study to estimate the prevalence of MS in the state [7, 15, 16].

## Methods

### Participant selection

Our study was an analysis of the Cardiological Society of India Kerala Chapter—CAD Risk Factors Prevalence (CSIK-CRP) study; the design and methods of this study has been previously published [17]. The CSIK-CRP study was a community based, cross-sectional population survey conducted from January to June 2011 in three major districts of Kerala state: Kozhikode in the north, Thrissur in the center, and Trivandrum in the south. Urban areas in Kerala are structured as municipal corporations that encompass the six major cities, along with municipalities that capture the surrounding suburban areas. Municipal corporations and municipalities are further divided into municipal wards each with a population size of 7000 to 15000. The Kozhikode, Thrissur and Trivandrum municipal corporations have 75, 55, and 100 municipal wards, respectively. For the urban sample, we randomly selected one municipal ward within each municipal corporation. We then utilized the 2010 voters’ list and identified the first 500 households. Rural areas in Kerala are structured as “grama panchayats” (for a population of 40,000 to 50,000), and these are further divided into panchayat wards. The Kozhikode, Trivandrum, and Thrissur districts have 75, 73, and 88 grama panchayats, respectively. There are between 6 to 13 wards in each panchayat. Initially, two grama panchayats were randomly selected from each districts. Later, one panchayat ward was randomly chosen from each of the selected panchayats. The survey was conducted among all households within the selected wards. The Kish method was used to select one individual age 20 to 59 years from each household, whereas all members age 60 to 79 years were selected.

Selected participants were sent a letter requesting participation. They were asked to visit an easily accessible facility with their medical records having fasted overnight. Non-responders were visited once at home and were encouraged to participate. The study was approved by the Ethics Committee of Cardiological Society of India, Kerala Chapter. Informed written consent was obtained from all participants.

Information on demographics, diet, physical activity, smoking, alcohol use, cardiac symptoms, personal and family history was collected using a structured survey questionnaire (S1 Table). The questionnaire was modeled off of several validated instruments including the WHO STEPS Instrument, the Rose Angina Questionnaire and the Minnesota Code Manual of Electrocardiographic findings, but was not validated prior to use [18–21]. Blood pressure (BP), waist circumference, lipid profile and fasting glucose measurements were performed. Participants were asked to rest for fifteen minutes prior to measurement of BP using an Omron model 1A2 electronic cuff (Omron Corporation, Shimogyo-ku, Kyoto, Japan). Three BP readings were taken three minutes apart and the mean of the last two readings was recorded as the BP. Waist circumference was measured at the midpoint between the iliac crest and the lower margin of the ribs using a non-stretchable measuring tape with participants standing erect in a relaxed position with both feet together. Blood samples for plasma glucose and lipid panel investigations were obtained from participants after 10 to 12 hours of fasting.

### Definitions and analysis

Demographic variables included age, sex and education. Physical activity was assessed through survey questions about participation in household chores and in leisure time physical activity. Participants were stratified for activity status based on the American Heart Association’s 2013 Guideline on Lifestyle Management to Reduce Cardiovascular Risk, which recommends that individuals engage in 150 minutes of moderate intensity physical activity per week to optimize cholesterol levels and blood pressure [22]. In our survey, participants were classified as inactive if they did not engage in leisure time physical activity, somewhat active if they exercised between 1 to 149 minutes per week, and adequately active if they exercised for 150 minutes or more per week. The American Heart Association 2000 Dietary Guidelines recommend increased intake of fruits, vegetables and whole grains with minimal consumption of saturated fats, salt, and alcohol [23]. Dietary habits were assessed through survey questions about overall dietary status (vegetarian vs. non-vegetarian), fruit and vegetable intake, fish consumption, oil consumption and intake of salty foods specific to Kerala.

The prevalence of MS, the individual diagnostic criteria for MS, associated diagnoses (hypertension, diabetes and hypercholesterolemia) and other potential risk factors were assessed, stratifying by sex and by urban-rural locality. Diagnostic criteria for metabolic syndrome are listed in Table 1. Three definitions (ATP III, IDF and Harmonization) were utilized so that results can be more easily compared with other studies. Hypertension was defined as a mean systolic or diastolic blood pressure of ≥140 mmHg or ≥90 mmHg, respectively, in two serial measurements taken three minutes apart, or as current use of antihypertensive medication. Diabetes was defined as a fasting glucose of ≥126mg/dL or current use of antidiabetic medication. Hypercholesterolemia was defined as a total cholesterol of ≥240 mg/dL or current use of lipid lowering medication. Body mass index categories were determined using the World Health Organization definition (underweight <18.5; normal 18.5–24.9; overweight 25–29.9; obese ≥30 kg/m^2^) [24]. Tobacco and alcohol use were presented in the format of never user, previous user, and current user. Family history of cardiovascular disease was defined as having a first degree relative with coronary artery disease or stroke.

Where specified, prevalence estimates underwent age standardization and adjustment for sex and urban-rural distribution. Age standardization was performed using the direct standardization approach, which produces a weighted average of stratum-specific rates using a standard reference population. We utilized the World Health Organization standard population for 2000–2025 aged 20 to 79 [25]. Each stratum consisted of a ten-year interval (for example, the first stratum consisted of individuals age 20 to 29) for a total of six strata. Age standardization was performed on the following subsets of study participants: rural men, rural women, urban men, and urban women. Once prevalence estimates were age standardized, population weights were applied for each subset using the Kerala 2011 census data on male-female and urban-rural distribution [26]. For example, the prevalence of ATP III MS in men was estimated by obtaining the age standardized rate for rural men and urban men, multiplying by the weights for urban men versus rural men in Kerala, and summing the two rates.

Proportions were compared using Pearson chi-square tests with significance levels set to a two-sided p<0.05. Univariate and multivariate logistic regression models were developed to identify participant characteristics that are associated with MS. Variables in the univariate model that correlated with MS at a relaxed 10% significance level (p<0.10) were included in the multivariate model. Alcohol, which is almost exclusively used by men, was excluded from the multivariate model because it was not applicable to both sexes.

The definition of definite CAD is based on any of the following [17]:

1. Documented evidence of prior acute coronary syndrome (ACS) or treatment for CAD
2. Documented history of undergoing coronary angioplasty or CABG
3. More than 50% epicardial coronary stenosis by invasive coronary angiography
4. ECG showing pathological Q waves (any of Minnesota code 1-1-1 to 1-1-7 or 1-2-1 to 1-2-5 or 1-2-7)
5. Imaging evidence of a region of loss of viable myocardium that is thinned and has a wall motion abnormality, in the absence of a non-ischemic cause
6. Rose Angina Questionnaire (RAQ) angina plus ECG changes (any of Minnesota codes 4-1-1, 4-1-2, 4–2 or 5-l, 5–2)
7. Rose Angina Questionnaire angina plus positive treadmill ECG (exercise-induced horizontal or down- sloping ST depression of ≥1 mm at 80 ms from J point) or inducible ischemia on stress imaging

The association between definite CAD and metabolic syndrome was determined in the form of unadjusted and adjusted odds ratios using univariate and multivariate logistic regression. Demographic variables that were significantly associated with CAD in a univariate model with p<0.10 were included in the multivariate model. Behavioral variables such as diet, exercise, tobacco and alcohol use were excluded from the multivariate model because they may not be stable over time. Patients often engage in behavior change following the diagnosis of CAD, thus behavioral variables may not be reliable correlates of disease.

We excluded individuals with incomplete or implausible data. We used Stata, version 14.1 (StataCorp, College Station, Texas, USA) for our analyses.

## Results

Of 5167 participants that were evaluated, 80 had missing values and 24 records were removed to exclude inaccurate waist circumference measurements. After these exclusions, we included 5063 participants for analysis.

### Demographic characteristics

The mean (SD) age of the participants was 51 (14) years. Three-fifths of participants were women (60% women vs. 40% men; Table 2). More than half of participants lived in rural areas (57% rural vs. 44% urban). Whereas 6% received no education, over half (53%) completed 10^th^ standard or above.

**Table 2:** Participant age and education status stratified by sex and geographic location.

	Rural Women		Urban Women		Rural Men		Urban Men		Total	
	(n = 1780)		(n = 1255)		(n = 1082)		(n = 946)		(n = 5063)	
	n	%	n	%	n	%	n	%	n	%
**Age**										
20–39	503	28.3	259	20.6	253	23.4	187	19.8	1202	23.7
40–59	787	44.2	560	44.6	412	38.1	423	44.7	2182	43.1
60–79	490	27.5	436	34.7	417	38.5	336	35.5	1679	33.2
**Education (years in school)**										
No education	148	8.3	69	5.5	45	4.2	16	1.7	278	5.5
Primary (1–4)	293	16.5	142	11.3	138	12.8	63	6.7	636	12.6
Secondary (5–9)	552	31.0	346	27.6	330	30.5	227	24.0	1455	28.7
Graduation (10)	485	27.2	369	29.4	327	30.2	328	34.7	1509	29.8
Junior college (11–12)	153	8.6	120	9.6	124	11.5	71	7.5	468	9.2
Senior college and beyond (13+)	149	8.4	209	16.7	118	10.9	241	25.5	717	14.2

### Metabolic syndrome prevalence

**Fig 1:**
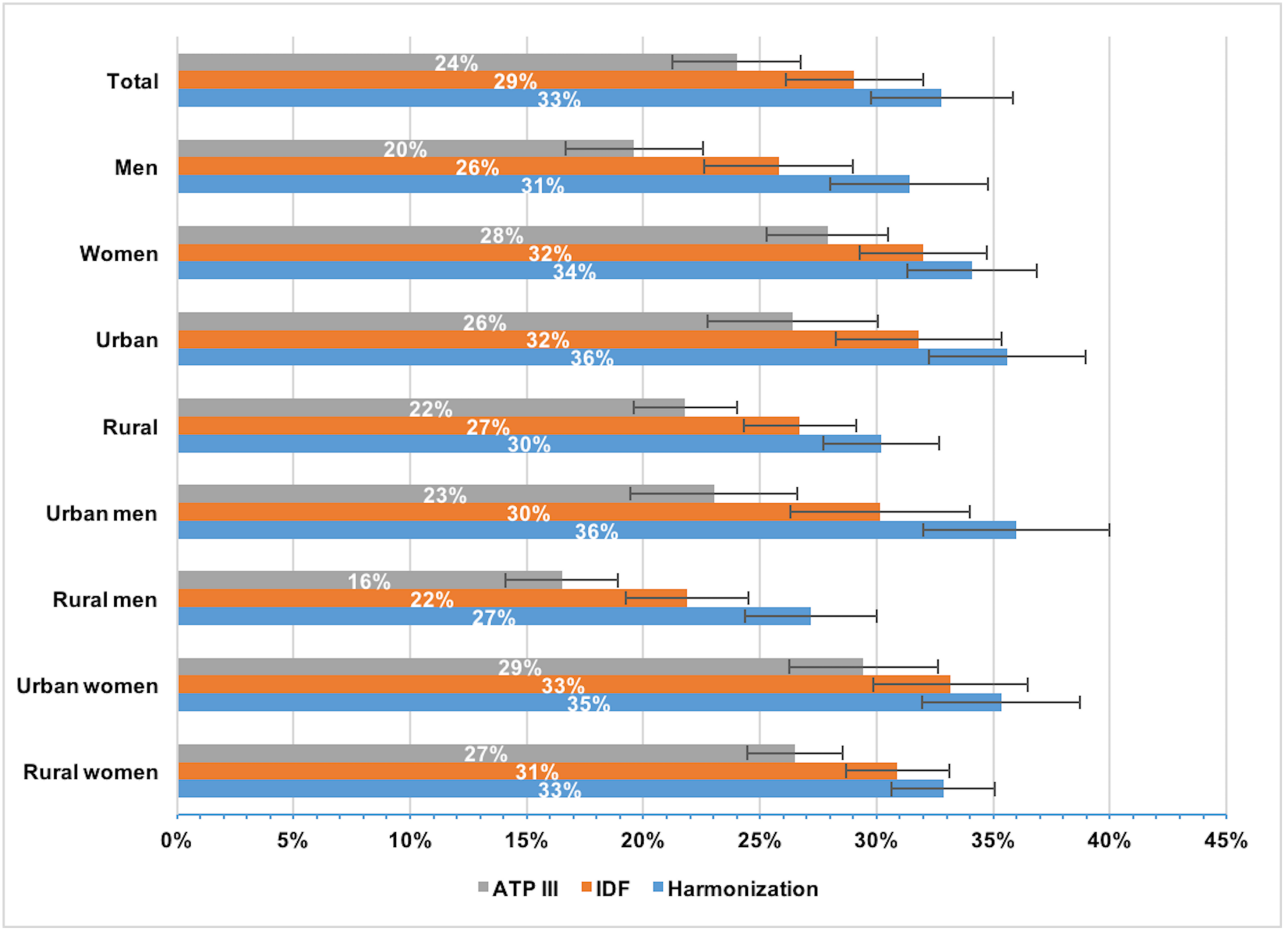
Age standardized prevalence of ATP III, IDF and Harmonization metabolic syndrome. Adjustment has been performed for sex and urban-rural location.

Following standardization for age and adjustment for sex and urban-rural distribution, the prevalence of metabolic syndrome was 24% for ATP III [95% CI: 21.3–26.8], 29% for IDF [26.1–32.1] and 33% for Harmonization [29.7–35.9] (Table 3 & Fig 1). Women had a higher prevalence of metabolic syndrome than men (ATP III: 20% men, 28% women, p<0.001), and individuals in urban areas had a higher prevalence than those in rural areas (ATP III: 26% urban, 22% rural, p<0.001).

**Table 3:** Crude and age standardized prevalence of metabolic syndrome using ATP III, IDF and Harmonization definitions.

	Rural women	Urban women	p-value	Rural men	Urban men	p-value	Rural^a^	Urban^a^	p-value	Women^a^	Men^a^	p-value	Total^a^
**ATP III**													
Crude	**32.9**	**37.6**		**20.1**	**25.3**		**26.9**	**31.9**		**35.2**	**22.5**		**29.3**
	30.8–35.1	35.0–40.3		17.8–22.6	22.6–28.1		24.7–29.2	29.2–34.6		32.8–37.6	20.1–25.2		26.8–31.8
Standardized	**26.5**	**29.4**	0.079	**16.5**	**23.0**	<0.001*	**21.8**	**26.4**	<0.001*	**27.9**	**19.6**	<0.001*	**24.0**
	24.5–28.6	26.2–32.6		14.1–18.9	19.5–26.6		19.6–24.0	23.1–29.8		25.3–30.5	16.7–22.6		21.3–26.8
**IDF**													
Crude	**37.5**	**41.8**		**26.3**	**33.8**		**32.2**	**38.1**		**39.5**	**29.9**		**35.0**
	35.2–39.7	39.0–44.5		23.8–29.1	30.9–36.9		29.9–34.7	35.2–41.0		37.1–42.0	27.2–32.8		32.4–37.7
Standardized	**30.9**	**33.2**	0.181	**21.9**	**30.2**	<0.001*	**26.7**	**31.8**	<0.001*	**32.0**	**25.8**	<0.001*	**29.1**
	28.7–33.1	29.8–36.5		19.3–24.5	26.3–34.0		24.2–29.1	28.2–35.3		29.2–34.7	22.6–29.0		26.1–32.1
**Harmonization**													
Crude	**40.4**	**44.8**		**33.0**	**41.1**		**36.9**	**43.1**		**42.5**	**36.9**		**39.9**
	38.1–42.7	42.0–47.5		30.3–35.9	38.0–44.3		34.4–39.5	40.2–46.0		40.0–45.0	34.0–39.9		37.2–42.6
Standardized	**32.9**	**35.3**	0.169	**27.2**	**36.0**	<0.001*	**30.2**	**35.6**	<0.001*	**34.1**	**31.4**	0.045*	**32.8**
	30.6–35.1	31.9–38.7		24.4–30.0	32.0–40.0		27.7–32.7	32.0–39.3		31.3–36.8	28.0–34.8		29.7–35.9

Age standardization was performed using the WHO standard population for 2000–2025. Percent prevalence is shown in bold with 95% confidence intervals below.

^a^ Population weights were added for urban-rural ratio and sex ratio using Kerala 2011 census data

### Hypertension, diabetes, and hypercholesterolemia prevalence

After standardization for age and adjustment sex and urban-rural distribution, the prevalence of hypertension was 29% [95% CI: 26.3–31.6] (Table 4). The prevalence of hypertension was higher in men than women (men 32%, women 26%, p<0.001). The overall prevalence of diabetes was 16% [13.5–17.6]. The prevalence of diabetes was also higher in men than women (men: 18%, women: 14%, p<0.001). The prevalence of hypercholesterolemia was 24% [21.1–26.4] with no gender differences (men: 25%, women 23%, p = 0.25). Urban areas demonstrated higher prevalence of hypertension (34% urban, 24% rural, p<0.001) and diabetes (17% urban, 14% rural, p = 0.005), while prevalence of hypercholesterolemia was not different (25% urban, 23% rural, p = 0.18).

**Table 4:** Age standardized prevalence of individual criteria for metabolic syndrome, related diagnoses and other potential risk factors among study participants.

		Rural women	Urban women	p-value	Rural men	Urban men	p-value	Women^a^	Men^a^	p-value	Total^a^
**Criteria for metabolic syndrome**	**Abdominal obesity**	**39.6**	**48.6**		**6.2**	**9.5**		**44.0**	**7.8**		**27.0**
	(ATP III)	37.0–42.2	44.7–52.6	<0.001*	4.6–7.8	6.9–12.1	0.005*	40.7–47.2	5.7–9.9	<0.001*	24.3–29.7
	**Abdominal obesity** (IDF & Harmonization)	**68.2**	**74.6**		**38.4**	**50.5**		**71.3**	**44.2**		**58.6**
		65.5–70.9	71.0–78.3	<0.001*	35.1–41.6	46.1–54.9	<0.001*	68.1–74.4	40.4–48.0	<0.001*	55.1–62.0
	**Low HDL Cholesterol**	**48.6**	**43.7**		**27.7**	**23.8**		**46.2**	**25.8**		**36.7**
		45.8–51.5	39.7–47.7	0.007*	24.3–31.0	20.0–27.6	0.046*	42.8–49.6	22.2–29.3	<0.001*	33.2–40.1
	**Hypertriglyceridemia**	**16.4**	**17.1**		**29.7**	**36.5**		**16.7**	**32.9**		**24.3**
		14.5–18.2	14.7–19.4	0.615	26.5–32.9	32.4–40.6	0.001*	14.6–18.8	29.3–36.6	<0.001*	21.5–27.1
	**Elevated blood pressure**	**32.2**	**39.6**		**42.2**	**57.7**		**35.8**	**49.6**		**42.2**
		30.1–34.2	36.5–42.7	<0.001*	38.8–45.6	53.3–62.1	<0.001*	33.2–38.3	45.7–53.5	<0.001*	39.1–45.4
	**Hyperglycemia**	**36.0**	**33.2**		**36.9**	**38.9**		**34.6**	**37.9**		**36.1**
		33.5–38.5	29.8–36.6	0.110	33.6–40.1	35.2–42.7	0.338	31.7–37.6	34.3–41.4	0.017*	32.9–39.3
**Related diagnoses**	**Hypertension**	**23.1**	**29.7**		**25.5**	**39.0**		**26.3**	**32.0**		**28.9**
		21.4–24.8	27.2–32.2	<0.001*	22.9–28.1	35.0–43.1	<0.001*	24.2–28.3	28.7–35.2	<0.001*	26.3–31.6
	**Diabetes**	**12.5**	**15.1**		**16.2**	**19.3**		**13.7**	**17.7**		**15.6**
		11.0–13.9	13.3–16.9	0.040	13.9–18.5	16.5–22.1	0.068	12.1–15.4	15.1–20.2	<0.001*	13.5–17.6
	**Hypercholesterolemia**	**22.6**	**23.7**		**23.4**	**25.6**		**23.1**	**24.5**		**23.8**
		20.7–24.5	21.4–25.9	0.473	20.4–26.4	22.0–29.3	0.250	21.0–25.2	21.2–27.8	0.251	21.1–26.4
**Other potential risk factors**	**BMI**										
	**Underweight**	**11.3**	**7.4**		**10.6**	**7.8**		**9.4**	**9.2**		**9.3**
		9.4–13.3	4.9–9.8		8.3–12.8	5.1–10.5		7.2–11.6	6.8–11.7		7.0–11.6
	**Normal**	**48.2**	**42.4**		**60.2**	**58.3**		**45.4**	**59.3**		**51.9**
		45.3–51.0	38.4–46.4		56.6–63.7	53.9–62.6		42.0–48.8	55.3–63.2		48.2–55.5
	**Overweight**	**31.3**	**36.2**		**24.5**	**27.2**		**S**	**25.8**		**30.0**
		28.8–33.8	32.5–40.0		21.4–27.5	23.6–30.8		30.6–36.8	22.4–29.1		26.8–33.2
	**Obese**	**9.0**	**13.8**		**4.8**	**6.8**		**11.3**	**5.7**		**8.7**
		7.5–10.5	11.1–16.6	<0.001*	3.2–6.5	4.3–9.2	<0.001*	9.2–13.4	3.7–7.8	<0.001*	6.6–10.8
	**Tobacco**										
	**Never**	**94.3**	**97.7**		**49.0**	**57.1**		**95.9**	**52.8**		**75.7**
		93.3–95.3	97.0–98.3		45.5–52.5	52.7–61.4		95.1–96.7	49.0–56.7		73.5–78.0
	**Previous** (no use in past month)	**0.9**	**0.9**		**16.0**	**9.2**		**0.9**	**12.8**		**6.5**
		0.5–1.2	0.5–1.3		13.8–18.3	6.9–11.5		0.5–1.3	10.5–15.1		5.2–7.7
	**Current**	**4.8**	**1.4**		**35.0**	**33.7**		**3.2**	**34.4**		**17.8**
		3.9–5.8	0.9–1.9	<0.001*	31.6–38.3	29.6–37.9	<0.001*	2.5–3.9	30.6–38.1	<0.001*	15.7–19.9
	**Alcohol**										
	**Never**	**99.8**	**99.9**		**47.6**	**46.8**		**99.9**	**47.2**		**75.2**
		99.6–100	99.9–100		44.0–51.2	42.5–51.1		99.7–100	43.3–51.2		73.3–77.1
	**Previous** (no use in past year)	**0.0**	**0.1**		**8.9**	**5.9**		**0.0**	**7.5**		**3.5**
			0.0–0.1		7.1–10.6	4.3–7.6			5.8–9.2		2.7–4.4
	**Monthly/Weekly**	**0.2**	**0.0**		**36.2**	**40.3**		**0.0**	**38.2**		**17.9**
		0.0–0.3			32.7–39.8	36.0–44.6			34.3–42.1		16.1–19.8
	**Daily**	**0.0**	**0.0**		**7.3**	**6.9**		**0.0**	**7.1**		**3.3**
				0.236	5.6–9.0	5.1–8.8	0.082		5.4–8.9	<0.001*	2.5–4.2
	**Family history** First degree relative with coronary artery disease or stroke	**21.3**	**25.2**		**22.9**	**23.2**		**23.2**	**23.1**		**23.1**
		19.1–23.5	22.2–28.2	0.012*	20.0–25.9	20.1–26.2	0.902	20.6–25.7	20.1–26.0	0.934	20.4–25.9

Displaying age adjusted prevalence (%) and 95% confidence interval.

^a^ Population weights were added for urban-rural ratio and sex ratio using Kerala 2011 Census Data.

### Prevalence of diagnostic criteria for metabolic syndrome

After standardization for age and adjustment for sex and urban-rural distribution, abdominal obesity prevalence was 27.0% [95% CI: 24.3–29.7] according to the ATP III definition and 58.6% [55.1–62.0] according to the IDF and Harmonization definitions (Table 4). One third of the population (36.7%) had low HDL cholesterol [33.2–40.1] and one quarter (24.3%) had elevated triglycerides [21.5–27.1]. Blood pressure was elevated in 42.2% [39.1–45.4] and glucose was elevated in 36.1% [32.9–39.3]. Women demonstrated a higher prevalence of abdominal obesity and low HDL (p<0.001 for both), whereas men demonstrated higher prevalence of hypertriglyceridemia, elevated blood pressure and hyperglycemia (p<0.02 for all). Individuals in urban areas demonstrated a higher prevalence of abdominal obesity (ATP III definition: 30.4% urban, 23.9% rural, p<0.001) and elevated blood pressure (48.1% urban, 36.9% rural, p<0.001) whereas individuals in rural areas had a higher prevalence of low HDL cholesterol (34.4% urban, 38.7% rural, p<0.05).

### Prevalence of other potential risk factors for metabolic syndrome

After standardization for age and adjustment for urban-rural distribution, the prevalence of current tobacco use was 34% for men and 3% for women (p<0.001; Table 4). Tobacco use was more prevalent in rural areas for both men and women (p<0.001). Few women engaged in alcohol use (<1%) compared with 38% for men.

In terms of family history, one quarter of participants (23%; standardized for age, and adjusted for urban-rural gradient and sex) reported a first degree relative with CAD or stroke.

We found that 34% of women and 26% of men were overweight (BMI 25.0–29.9 kg/m^2^) and 11% of women and 6% of men were obese (BMI≥30 kg/m^2^). Women had significantly higher BMI than men (p<0.001), and individuals in urban areas had significantly higher BMI compared to those living in rural areas (p<0.001).

The majority of women (95% adjusted prevalence) engaged in household chores such as sweeping, mopping and clothes washing, and close to half of men (46%) did so as well (S2 Table). Two-fifths of women engaged in leisure time physical activity (20%) compared to half of men (52%). Women mostly engaged in walking (17% of all women) and active outdoor work (2%), whereas men mostly engaged in walking (34% of all men), outdoor games (13%) and cycling (8%). More men exercised 150 or more minutes per week compared to women (36% versus 11%, p<0.001). Similarly, more urban residents engaged in leisure time physical activity compared to rural residents (27% versus 19% exercise ≥150 minutes per week, p<0.001).

Most participants (95% adjusted prevalence) were non-vegetarian and utilized fish, rice and coconut-based products as staples in the diet. Participants consumed a median of 7 servings of fish per week (interquartile range 3 to 7). Most (93%) used coconut oil for cooking. A minority (3%) consumed 5 or more servings of fruits and vegetables a day with a sample median of 2.1 servings daily (interquartile range 1.4 to 2.9). Two-thirds ate pickle (62%) and papad (65%) at least once a week, and one-third ate salted rice (31%) or salted fish (34%) at least once a week.

### Logistic regression models to determine metabolic syndrome risk factors

The univariate logistic regression model (Table 5) revealed that the odds of ATP III MS are higher among women (OR 1.84, 95% CI 1.62–2.19, p<0.001), urban inhabitants (OR 1.22, 95% CI 1.08–1.38, p = 0.001), and among older individuals (OR 1.16 for 5-year increments, 95% CI 1.14–1.19, p<0.001). Prevalence of MS was three and six times higher in overweight [OR: 2.73; 2.38–3.14] and obese individuals [OR: 6.26; 5.04–7.76], respectively, as compared to individuals with normal BMI (p<0.001 for both). Individuals who completed grade 10 and above had a lower likelihood of developing MS compared to those without education (OR 0.85 for 4-year increments, 95% CI 0.81–0.90, p<0.001). The propensity for MS was 21% lower in current tobacco users as compared to never users (OR 0.79, 95% CI 0.67–0.93, p = 0.005). Interestingly, greater fruit and vegetable intake was associated with higher propensity for MS, with individuals taking 5 or more servings having an OR of 1.42 compared to those taking 0 to 1 servings daily (95% CI 1.01–2.00; p = 0.04). Among men, a history of previous alcohol use, defined as positive history of alcohol use but with no drinks in the past year, increased the odds of MS compared to never users (OR 1.42, 95% CI 1.01–2.01, p = 0.044), whereas regular drinking did not have an effect. Family history and physical activity status did not have any association with MS.

**Table 5:** Risk factors associated with ATP III metabolic syndrome in a univariate logistic regression model.

	Cases of ATP III MS/total	% crude prevalence of ATP III MS	Odds Ratio	95% Confidence Interval		p-value
Sex						
Male	456/2028	22.5	1			
Female	1058/3035	34.9	1.84	1.62	2.10	<0.001*
Geographic location						
Rural	803/2862	28.1	1			
Urban	711/2201	32.3	1.22	1.08	1.38	0.001*
Alcohol use (male only)						
Never used	204/923	22.1	1			
Past use (no drinks in past year)	57/198	28.8	1.42	1.01	2.01	0.044*
Monthly or weekly use	156/732	21.3	0.95	0.75	1.21	0.699
Daily use	39/175	22.3	1.01	0.69	1.49	0.957
Tobacco use						
Never used	1171/3778	31.0	1			
Past use (no use in last month)	112/403	27.8	0.86	0.68	1.08	0.185
Current use	231/882	26.2	0.79	0.67	0.93	0.005*
Family history of CAD or stroke						
No	1121/3772	29.7	1			
Yes	393/1291	30.4	1.03	0.90	1.19	0.625
BMI						
Underweight (<18.5)	42/457	9.2	0.39	0.28	0.54	<0.001*
Normal (18.5–24.9)	532/2563	20.8	1			
Overweight (25.0–29.9)	668/1601	41.7	2.73	2.38	3.14	<0.001*
Obese (30 +)	272/438	62.1	6.26	5.04	7.76	<0.001*
Fruits and vegetables (servings per day)						
0 to 1	482/1723	28.0	1			
2 to 4	974/3177	30.7	1.14	1.00	1.30	0.050
5 or more	57/160	35.6	1.42	1.01	2.00	0.041*
Engagement in leisure time physical activity						
None	1053/3457	30.5	1			
1–149 minutes per week	153/509	30.1	0.98	0.80	1.20	0.854
≥150 minutes per week	308/1097	28.1	0.89	0.77	1.04	0.133
Education						
None	106/278	38.1	1			
Primary (1–4)	206/636	32.4	0.78	0.58	1.04	0.093
Secondary (5–9)	481/1455	33.1	0.80	0.61	1.05	0.102
Graduation (10)	436/1509	28.9	0.66	0.51	0.86	0.002*
Junior college (11–12)	107/468	22.9	0.48	0.35	0.67	<0.001*
Senior college and above (13+)	178/717	24.8	0.54	0.40	0.72	<0.001*
Education (4 year increments)	-	-	0.85	0.81	0.90	<0.001*
Age						
20–29	37/324	11.4	1			
30–39	145/878	16.5	1.53	1.04	2.26	0.030*
40–49	331/1179	28.1	3.03	2.10	4.36	<0.001*
50–59	371/1003	37.0	4.55	3.16	6.56	<0.001*
60–69	447/1189	37.6	4.67	3.25	6.71	<0.001*
70–79	183/490	37.3	4.62	3.14	6.82	<0.001*
Age (5 year increments)	-	-	1.16	1.14	1.19	<0.001*

In the multi-variate analyses (Table 6), overweight and obesity showed stronger association with ATP III metabolic syndrome with relatively large effect sizes (OR 3.01 and 7.18 for overweight and obese BMI, respectively, compared to normal). Other variables associated with MS were female sex (OR 1.77, 95% CI: 1.49–2.11, p<0.001), and older age (OR 1.22 for 5 year increments, 95% CI: 1.19–1.26, p<0.001). Geographic location, years of education, tobacco use status, and fruit and vegetable intake were not associated with MS in the multivariate model.

**Table 6:** Risk factors associated with ATP III metabolic syndrome in a multivariate logistic regression model.

	Odds Ratio	95% Confidence Interval		p-value
Sex				
Male	1			
Female	1.77	1.49	2.11	<0.001*
Geographic location				
Rural	1			
Urban	1.06	0.92	1.22	0.434
Age (5 year increments)	1.22	1.19	1.26	<0.001*
Education (4 year increments)	0.94	0.89	1.01	0.072
BMI				
Underweight	0.31	0.22	0.44	<0.001*
Normal	1			
Overweight	3.01	2.6	3.49	<0.001*
Obese	7.18	5.71	9.04	<0.001*
Tobacco use				
Never use	1			
Past use	1.1	0.83	1.45	0.510
Current use	1.23	1	1.52	0.054
Fruits and vegetables (servings per day)				
0 to 1	1			
2 to 4	1.09	0.94	1.27	0.231
5 or more	1.3	0.89	1.89	0.177

### Association with coronary artery disease

The crude prevalence of definite CAD was 8.3% for ATP III, 7.6% for IDF and 7.8% for Harmonization (Table 7). ATP III metabolic syndrome had a strong association with definite CAD in the univariate logistic regression model (unadjusted OR 1.89, 95% CI 1.48–2.40, p<0.001) and the strongest association in the multivariate logistic regression model (adjusted OR 1.69, 95% CI 1.31–2.18, p<0.001), which adjusted for sex, urban-rural status, age, education and family history of CAD or stroke.

**Table 7:** Crude prevalence, unadjusted and adjusted odds ratios for definite coronary artery disease among participants with metabolic syndrome.

	MS cases with definite CAD/total MS cases	% of MS participants with definite CAD	Unadjusted odds ratio for definite CAD	95% Confidence Interval		p-value	Adjusted odds ratio for definite CAD^a^	95% Confidence Interval		p-value
ATP III	126/1514	8.3%	1.89	1.48	2.40	<0.001*	1.69	1.31	2.18	<0.001*
IDF	137/1796	7.6%	1.69	1.33	2.15	<0.001*	1.54	1.20	1.98	0.001*
Harmonization	159/2027	7.8%	1.90	1.50	2.42	<0.001*	1.57	1.22	2.01	<0.001*

^a^ Adjusted for sex, urban vs. rural location, age, education and family history through a multivariate logistic regression model

## Discussion

In a cross-sectional study conducted in three districts in Kerala, India, covering over 5000 participants, 1 of 4 individuals had ATP III metabolic syndrome (24%). The prevalence estimates were even higher when using the IDF and Harmonization definitions (29% and 33%, respectively). Female sex, BMI and age were independent predictors of MS in this population. Metabolic syndrome was associated with higher propensity for the presence of definite CAD.

Over the past few decades, the state of Kerala has experienced a rising burden of CAD and cardiovascular mortality [8,9]. Heart attacks were the leading cause of death in 2015, accounting for 27% of deaths in the state [27]. The high prevalence of metabolic syndrome may be contributing to the large burden of CAD in Kerala. Individuals with MS in our study had nearly double the odds of having CAD compared to those without MS. The relatively strong association of MS with definite CHD in this population is not surprising as MS is known to double the risk of developing cardiovascular disease in a 5 to 10-year period [6]. The ATP III definition was a better predictor of CAD than the IDF and Harmonization definitions, suggesting that this definition may have greater clinical utility in this region.

Based on our findings, the Kerala population appears to have a relatively high burden of MS compared to neighboring states and countries. A systematic review of MS prevalence in South Asia found age adjusted rates of IDF MS starting from 15% in the Philippines and 18% in China, and as high as 35% in Pakistan [28]. Prabhakaran et al (2006) found a large urban-rural difference in MS in Delhi and the surrounding rural areas for the time period 1991 to 1995 [29]. However, our study demonstrated a less pronounced urban-rural gradient in Kerala. The diminished urban-rural difference may be attributable to the unique situation in Kerala, where almost all rural areas have become urbanized, and also due to Kerala’s progress along the epidemiological transition over the past two decades [7].

The prevalence of MS in Kerala was higher among women, older individuals, and those with greater body mass index in the multivariate model, which are established trends globally. Overweight and obesity had the greatest correlation, increasing the risk of MS by 3 and 7 times, respectively. The prevalence of metabolic syndrome decreased with higher levels of education. The crude prevalence of MS was one-third in individuals who attended nine years of school or less, compared to one-fourth in those who completed twelve years and above. This finding suggests a need for lifestyle interventions targeting individuals with fewer years of schooling.

Clinical guidelines for managing MS focus on lifestyle modifications with an emphasis on diet and exercise interventions [30]. Our dietary assessment suggested that the population consumed large quantities of fish and limited fruits and vegetables. A more thorough dietary characterization in which caloric consumption and overall rice intake is taken into account may shed light on the scope for dietary interventions. However, we found a gender gap in engagement in leisure time physical activity. Half of men exercised at least once a week compared to only one out of five women. Interestingly, engagement in physical activity did not correlate with MS prevalence in the univariate model. This may be because the population did not engage in exercise of high enough intensity, or perhaps because individuals begin exercising as a response to deteriorating health. A more thorough characterization of physical activity based on objective measurements are required to delineate the relationship between physical activity and MS. Studies have found that more walkable cities allow for greater activity throughout the day, particularly for females. In fact, the prevalence of obesity decreases more rapidly in females than in males as step volume increases [31]. Walkability studies and step volume data in Kerala may be of great value to understand the environmental contributors of metabolic syndrome and to decrease the gender gap.

### Limitations

Utilization of cross-sectional data limits our ability to assess causality. The survey relied on self-reporting for demographic and behavioral variables, which potentially led to underreporting in certain variables. The survey approach led to overrepresentation of the available members of each household at the time of survey. For example, women, the elderly, and rural inhabitants were overrepresented. We tried to correct for this by presenting disaggregated data, by performing age adjustments using the WHO standard population, and by according weights based on the sex ratio and urban-rural ratio using the 2011 Kerala census data. Another limitation was the assessment of diet and physical activity. We did not use validated tools, which limits our ability to draw conclusions or compare our findings to other studies.

## Conclusions

This study highlights the relatively high prevalence of MS in the Kerala population and its association with CAD. The adjusted prevalence of MS was 24% for ATP III criteria, 29% of IDF, and 33% for Harmonization. Individuals with ATP III MS had almost two times the odds for having definite coronary artery disease as compared to individuals without MS. Overweight or obese body mass index, female sex, and urban residence were strongly associated with MS. Future studies should investigate MS incidence and shed further light on the influence of dietary and physical activity practices of the Kerala population on MS. The prevalence of MS is particularly high in women, individuals living in urban areas, and individuals with fewer years of schooling. Policy makers and healthcare providers can develop effective and equitable interventions for primordial and primary prevention by addressing the risk factors for MS among high-risk populations as part of the strategy for cardiovascular health improvement.

## Supporting information

S1 Table Survey questionnaire. The questionnaire that was administered to study participants is available for reference.
(PDF)

S2 Table Physical activity and diet characterization of participants. Prevalence estimates have been standardized for age and adjusted for sex and urban-rural distribution (% and 95% confidence interval).
(PDF)
