# Supplementary material for: Prevalence of metabolic syndrome and its risk factors in Kerala, South India: Analysis of a community based cross-sectional study
Source: PLoS One. 2018 Mar 27;13(3):e0192372. doi: 10.1371/journal.pone.0192372 (PMC5870937; doi:10.1371/journal.pone.0192372)
Supplement: S1 Table — The questionnaire that was administered to study participants is available for reference. (PDF) [file pone.0192372.s001.pdf]

CSI Kerala CRP Study Questionnaire

Identification Information (Section I)		
I 1	Center code	☐
I 2	Respondent Identification Number	☐☐☐☐
I 3	House number	☐☐ / ☐☐☐☐ / ☐
I 4	Household Identification Number	☐☐☐☐
I 5	Interviewer code	☐☐☐
I 6	Date of administration of the questionnaire	☐☐ / ☐☐ / ☐☐☐☐<br>Day Month Year

		Respondent Id Number ☐☐☐☐
	**Consent**	
I 7	Consent has been obtained? (written)	Yes 1<br>No 2<br>If No, record only age & sex of respondent
I 8	Time of interview (24 hour clock)	☐☐ : ☐☐
I 9	Name (Block letters)	
I 10	Complete residential address	
	House Name	
	Full Address	
	Post Office	
	Pin	☐☐☐☐☐☐
	District	
I 11	Contact phone numbers with STD code	☐☐☐☐☐☐☐☐☐☐☐<br>☐☐☐☐☐☐☐☐☐☐☐

Respondent identification Number

Demographic Information (Section C) ജനനസ്ഥിതിവിവര പഠനം (സെക്ഷൻ സി)

**C 1**	Gender (Record Male / Female as observed)	Male<br>Female	1<br>2
**C 2**	Date of birth<br>If don't know, go to C3	Day Month Year	
**C 3**	Age	Years	
**C 4**	Marital status	Married<br>Not married<br>Widow<br>Seperated<br>Divorced	1<br>2<br>3<br>4<br>5
**C 5**	What is your main work status for the last one year?<br>കഴിഞ്ഞ ഒരു കൊല്ലത്തെ നിങ്ങളുടെ മുഖ്യ ജോലി ഏതു തരത്തിലുള്ളതാണ്?	Professional/Executive/ Big business<br>Clerical/ medium business<br>Self-employed/ skilled<br>Unskilled/ landless labourer<br>Student<br>Homemaker<br>Retired<br>Unemployed (able to work)<br>Unemployed (unable to work)<br>Others	01<br>02<br>03<br>04<br>05<br>06<br>07<br>08<br>09<br>10
**C 6**	Please specify your exact occupation for the last one year<br>കഴിഞ്ഞ ഒരു കൊല്ലത്തെ നിങ്ങളുടെ കൃത്യമായ തൊഴിൽ എന്തെന്ന് പ്രത്യേകം എടുത്തു പറയുക.		
**C 7**	How many years of formal education did you complete<br>എത്രകൊല്ലത്തെ ഔപചാരിക വിദ്യാഭ്യാസം നിങ്ങൾ പൂർത്തിയാക്കിയിട്ടുണ്ട്?		
**C 8**	What is the total number of adults in your household ?<br>നിങ്ങളുടെ വീട്ടിലെ പ്രായപൂർത്തിയായവരുടെ മൊത്തം എണ്ണമെത്ര?		
**C 9**	Do you possess BPL ration card ?<br>നിങ്ങൾക്ക് ബി പി എൽ റേഷൻ കാർഡുണ്ടോ?	Yes<br>No.	1<br>2
**C 10**	Religion<br>മതം	Christianity<br>Hinduism<br>Islam<br>Atheist/ others	01<br>02<br>03<br>04
**C 11**	Caste<br>ജാതി	Forward<br>Backward<br>Sc / ST	01<br>02<br>03
**C 12**	Type of family<br>ഏതുതരം കുടുംബമെന്ന്	Nuclear<br>Joint / Extended	01<br>02

Respondent identification Number

Standard of Living Index NFHS (Section L)					
	**Household characterestics**	**Scores**			
L 1	House type	pucca - 4	semi pucca = 2	kachha = 0	
L 2	Separate room for cooking	Yes = 1	No = 0		
L 3	Ownership of house	Yes = 1	No = 0		
L 4	Toilet facility	own flush toilet = 4	public or shared flush toilet or own pit toilet = 2	shared or public pit toilet = 1	no facility = 0
L 5	Source of lighting	electricity = 2	kerosene, gas, oil = 1	other source of lighting = 0	
L 6	Main fuel for cooking	electricity, liquid petroleum gas or biogas = 2	coal, charcoal or kerosene = 1	other fuel = 0	
L 7	Source of drinking water	pipe, hand pump, well in residence / yard / plot = 2	public tap, hand pump or well = 1	other water source = 0	
L 8	Car or tractor	Yes = 4	No =0		
L 9	Moped or scooter	Yes = 3	No =0		
L 10	Telephone	Yes = 3	No =0		
L 11	Refrigerator	Yes = 3	No =0		
L 12	Colour TV	Yes = 3	No =0		
L 13	Black and white TV	Yes = 2	No =0		
L 14	Bicycle	Yes = 2	No =0		
L 15	Electric fan	Yes = 2	No =0		
L 16	Radio	Yes = 2	No =0		
L 17	Sewing machine	Yes = 2	No =0		
L 18	Mattress	Yes = 1	No =0		
L 19	Pressure cooker	Yes = 1	No =0		
L 20	Chair	Yes = 1	No =0		
L 21	Cot or bed	Yes = 1	No =0		
L 22	Table	Yes = 1	No =0		
L 23	Clock or watch	Yes = 1	No =0		
L 24	Ownership of livestock	Yes = 2	No =0		
L 25	Water pump	Yes = 2	No =0		
L 26	Bullock cart	Yes = 2	No =0		
L 27	Thresher	Yes = 2	No =0		
Circle the appropriate number					

Respondent identification Number ☐☐☐☐

**Behavioral Measures** പെരുമാറ്റരീതിയെ സംബന്ധിച്ച വിവരങ്ങൾ				
**Tobacco Use (Section S)** പുകയില ഉപയോഗം (സെക്ഷൻ എസ്)				
**S 1**	Have you ever used tobacco products in life ?<br>ജീവിതത്തിൽ എന്നെങ്കിലും നിങ്ങൾ പുകയില ഉൽപന്നങ്ങൾ ഉപയോഗിച്ചിട്ടുണ്ടോ?	Yes	1	
		No	2	*Skip to A 1*
**S 2**	Have you used the following tobacco products in last one month ?<br>കഴിഞ്ഞ ഒരു മാസത്തിൽ താഴെ പറയുന്ന പുകയില ഉല്പന്നങ്ങൾ നിങ്ങൾ ഉപയോഗിച്ചിട്ടുണ്ടോ?<br>(Multiple response)<br>Record all responses	Beedi<br>Cigarette<br>Snuff<br>Chewing<br>None	A<br>B<br>C<br>D<br>E	*Skip to S 5*
**S 3**	How old were you when you first started smoking ?<br>പുകവലി ആദ്യമായി ആരംഭിച്ചപ്പോൾ നിങ്ങൾക്ക് എത്ര വയസ്സായിരുന്നു?	☐☐	(Code 99 if don't know)	
**S 4**	Quantity of tobacco use per day, week or month<br>ദിവസമോ ആഴ്ചയോ മാസമോ ഉള്ള പുകയില ഉപയോഗത്തിന്റെ അളവ്	(CODE 99 FOR DON'T KNOW or DON'T REMEMBER)		
**S 4 a**	Beedi<br>ബീഡി	Daily 1<br>Weekly 2<br>Monthly 3	Numbers / day ☐☐<br>Numbers / week ☐☐<br>Numbers / month ☐☐	
**S 4 b**	Cigarette<br>സിഗരറ്റ്	Daily 1<br>Weekly 2<br>Monthly 3	Numbers / day ☐☐<br>Numbers / week ☐☐<br>Numbers / month ☐☐	
**S 4 c**	Snuff<br>മൂക്കുപൊടി	Daily 1<br>Weekly 2<br>Monthly 3	Numbers / day ☐☐<br>Numbers / week ☐☐<br>Numbers / month ☐☐	
**S 4 d**	Chewable tobacco<br>ചവയ്ക്കുന്ന പുകയില	Daily 1<br>Weekly 2<br>Monthly 3	Numbers / day ☐☐<br>Numbers / week ☐☐<br>Numbers / month ☐☐	

Respondent identification Number ☐☐☐☐

Behavioral Measures

Tobacco Use (Section S)

S 5	If not used tobacco products in the last one month, how long ago did you stop smoking / smokeless tobacco<br>കഴിഞ്ഞ ഒരു മാസമായി പുകയില ഉല്പന്നങ്ങൾ ഉപയോഗിച്ചിട്ടില്ലെങ്കിൽ എത്രകാലം മുമ്പാണ് നിങ്ങൾ പുകവലി / പുകയില്ലാത്ത പുകയില നിർത്തിയത്?	Weeks ago 1<br>Months ago 2<br>Years ago 3	Number of weeks ☐☐<br>Number of months ☐☐<br>Number of years ☐☐
S 6	What were the kinds of tobacco products you were using before ?<br>മുമ്പ് ഏതൊക്കെ തരത്തിലുള്ള പുകയില ഉല്പന്നങ്ങളാണ് നിങ്ങൾ ഉപയോഗിച്ചുകൊണ്ടിരുന്നത്?<br>(Multiple response)<br>Record all responses	Beedi<br>Cigarette<br>Snuff<br>Chewing	A<br>B<br>C<br>D

Alcohol Consumption (Section A) മദ്യോപയോഗം (സെക്ഷൻ എ)

A 1	Have you ever consumed a drink that contains alcohol such as beer, whisky, rum, gin, brandy, wine, toddy or arrack?<br>ബിയർ, വിസ്കി, റം, ജീൻ, ബ്രാണ്ടി, വൈൻ, കള്ള്, ചാരായം പോലുള്ള ആൽക്കഹോൾ അടങ്ങിയ മദ്യം നിങ്ങൾ എപ്പോഴെങ്കിലും ഉപയോഗിച്ചിട്ടുണ്ടോ?	Yes 1<br>No. 2	 → *Skip to P 1*
A 2	Have you consumed alcohol within the past 12 months?<br>കഴിഞ്ഞ 12 മാസങ്ങൾക്കുള്ളിൽ നിങ്ങൾ ആൽക്കഹോൾ ഉപയോഗിച്ചിട്ടുണ്ടോ?	Yes 1<br>No. 2	 → *Skip to A 5*
A 3	In the past 12 months, how frequently have you had at least one drink?<br>കഴിഞ്ഞ 12 മാസങ്ങളിൽ നിങ്ങൾ എത്ര തവണ ചുരുങ്ങിയത് ഒരു ഡ്രിങ്ക് എങ്കിലും കഴിച്ചിട്ടുണ്ടായിരിക്കും ?	Daily 1<br>Weekly 2<br>Monthly 3	Number / day ☐☐<br>Number / week ☐☐<br>Number / month ☐☐
A 4	When you drink alcohol, on average, how many drinks do you have during one day?<br>നിങ്ങൾ മദ്യപിക്കുമ്പോൾ ഒരു ദിവസം ശരാശരി എത്രതവണ മദ്യം കഴിക്കും?	Number ☐☐	*Don't know 99*
A 5	When you drink alcohol, how much time do you spend for drinking?<br>മദ്യപിക്കുമ്പോൾ കുടിക്കാൻ എത്രസമയം നിങ്ങൾ ചെലവഴിക്കുന്നു?	Minutes 1<br>Hours 2	Time in minutes ☐☐<br>Time in hours ☐☐
A 6	If not consumed alcohol within past 12 months, how long ago did you stop alcohol ?<br>കഴിഞ്ഞ 12 മാസമായി നിങ്ങൾ മദ്യപിച്ചിട്ടില്ലെങ്കിൽ, എത്രകാലം മുമ്പാണ് നിങ്ങൾ മദ്യപാനം നിർത്തിയത്?	Months ago 1<br>Year ago 2	Number of months ☐☐<br>Number of Year ☐☐

Respondent identification Number ☐☐☐☐

**Physical Activity (Section P)** ശാരീരിക അദ്ധ്വാനം (സെക്ഷൻ പി)			
**P 1**	Does your work outside home involve physical activity?<br>വീട്ടിനുപുറത്തെ നിങ്ങളുടെ ജോലി ശാരീരിക അദ്ധ്വാനം ഉൾപ്പെടുന്നതാണോ?	Yes 1 *Go to P 2*<br>No. 2 *Go to P 3*	
**P 2**	What kind of work you do which involve physical activity ?<br>ശാരീരിക അദ്ധ്വാനം ഉൾപ്പെടുന്ന ഏതുതരം ജോലിയാണ് നിങ്ങൾ ചെയ്യുന്നത്?	*Specify*	
**P 3**	Do you engage in household chores ?<br>വീട്ടുജോലികളിൽ നിങ്ങൾ ഏർപ്പെടാറുണ്ടോ?	Yes 1 *Go to P 4*<br>No. 2 *Go to P 5*	

**P 4**	What kind of household chores do you do ?<br>ഏതൊക്കെതരം വീട്ടുജോലികളാണ് നിങ്ങൾ ചെയ്യുന്നത്?<br>(Multiple response)<br>Record all responses		
		Washing of cloths A	Number of days per week ☐
		Sweeping house B	Number of days per week ☐
		Mopping house C	Number of days per week ☐
		Cleaning premises D	Number of days per week ☐
		Drawing water from well E	Number of days per week ☐

**P 5**	Do you indulge in the following activities ?<br>താഴെ പറയുന്ന പ്രവർത്തനങ്ങളിൽ നിങ്ങൾ ഏർപ്പെടാറുണ്ടോ?<br>(Multiple response)<br>Record all responses		Time in minutes	Number of days/week
		Outdoor games A	☐☐	☐
		Use of cycle B	☐☐	☐
		Treadmill C	☐☐	☐
		Walking D	☐☐	☐
		Active out door work E	☐☐	☐
		None of the above F	☐☐	☐

Diet (Section D) ആഹാരരീതി (സെക്ഷൻ ഡി)

**D 1**	Dietary pattern<br>ആഹാരരീതി	Vegetarian<br>Vegetarian plus egg<br>Mixed diet	1<br>2<br>3	 *Skip to D 4*
**D 2**	How many servings of fish do you consume each week ?<br>ഓരോ ആഴ്ചയും എത്രതവണ നിങ്ങൾ മത്സ്യം കഴിക്കും?			☐☐
**D 3**	How many servings of salted fish do you consume each week ?<br>ഓരോ ആഴ്ചയും എത്ര തവണ നിങ്ങൾ ഉപ്പിട്ട മത്സ്യം കഴിക്കും?			☐☐

D 4	In a common week, on how many days do you eat fruit ?<br>ഒരു സാധാരണ ആഴ്ചയിൽ എത്രദിവസമാണ് നിങ്ങൾ പഴവർഗ്ഗങ്ങൾ കഴിക്കുന്നത്?	Number of days	
D 5	How many servings of fruit do you eat on one of those days ?<br>ആ ദിവസങ്ങളിലൊന്നിൽ എത്രതവണ നിങ്ങൾ പഴവർഗ്ഗങ്ങൾ കഴിക്കും?	Number of servings	
D 6	In a common week, on how many days do you eat vegetables ?<br>ഒരു സാധാരണ ആഴ്ചയിൽ എത്രദിവസം നിങ്ങൾ പച്ചക്കറികൾ കഴിക്കും?	Number of days	
D 7	How many servings of vegetables do you eat on one of those days ?<br>ആ ദിവസങ്ങളിലൊന്നിൽ എത്രതവണ നിങ്ങൾ പച്ചക്കറികൾ കഴിക്കും?	Number of servings	
D 8	What type of oil or fat is most often used for meal preparation in your household ?<br>നിങ്ങളുടെ വീട്ടിൽ ഭക്ഷണം പാകം ചെയ്യുന്നതിന് ഏതുതരം എണ്ണ അഥവാ കൊഴുപ്പ് ആണ് ഏറ്റവുമേറെ ഉപയോഗിക്കാറുള്ളത്?	Coconut oil A<br>Palm oil B<br>Sunflower oil C<br>Refined vegetable oil D<br>Gingely oil E<br>Olive oil F<br>other G	*If other specify the oil*
D 9	Do you follow the practice of adding salt to rice when being cooked or served ?<br>പാകം ചെയ്യുമ്പോഴോ വിളമ്പുന്ന സമയത്തോ ചോറിൽ ഉപ്പുചേർക്കുന്ന രീതി നിങ്ങൾ പിന്തുടരാറുണ്ടോ?	Yes<br>No.	1<br>2
D 10	How many servings of pickle / pappad do you consume each week?<br>ഓരോ ആഴ്ചയും ഭക്ഷണത്തോടൊപ്പം നിങ്ങൾ എത്രതവണ അച്ചാറ് / പപ്പടം കഴിക്കും?	Pickle 1 Number of days/week ☐ servings / Day ☐<br>Pappad 2 Number of days/week ☐ servings / Day ☐	

Respondent identification Number ☐☐☐☐

Family History of CAD, Stroke, Diabetes, Hypertension (Section F)
സി എ ഡി, പക്ഷാഘാതം, പ്രമേഹം, രക്താതിസമ്മർദ്ദം എന്നിവ സംബന്ധിച്ച കുടുംബ ചരിത്രം (സെക്ഷൻ എഫ്)

**F 1**	Do any of your first degree relatives have evidence of CAD?<br>നിങ്ങളുടെ ഒന്നാം തലമുറ ബന്ധുക്കളിൽ ആർക്കെങ്കിലും സി എ ഡി യുടെ ലക്ഷണമുണ്ടോ?	Yes<br>No	1<br>2 Don't know
**F 2**	Do any of your first degree relatives have history of Stroke?<br>നിങ്ങളുടെ ഒന്നാം തലമുറ ബന്ധുക്കളിൽ ആർക്കെങ്കിലും പക്ഷാഘാതം ചരിത്രമുണ്ടോ?	Yes<br>No	1<br>2 Don't know
**F 3**	Do any of your first degree relatives have Diabetes?<br>നിങ്ങളുടെ ഒന്നാം തലമുറ ബന്ധുക്കളിൽ ആർക്കെങ്കിലും പ്രമേഹമുണ്ടോ?	Yes<br>No	1<br>2 Don't know
**F 4**	Do any of your first degree relatives have Hypertension?<br>നിങ്ങളുടെ ഒന്നാം തലമുറ ബന്ധുക്കളിൽ ആർക്കെങ്കിലും രക്താതിസമ്മർദ്ദമുണ്ടോ?	Yes<br>No	1<br>2 Don't know
**F 5**	Do any of your first degree relatives have Hypercholesterolemia?<br>നിങ്ങളുടെ ഒന്നാം തലമുറ ബന്ധുക്കളിൽ ആർക്കെങ്കിലും ഹൈപർ കൊളസ്റ്ററോളീമിയ (രക്തം കട്ടപിടിക്കുന്നതിനു ഹേതുവായി കരുതപ്പെടുന്ന കൊഴുപ്പിന്റെ ആധിക്യം) ഉണ്ടോ?	Yes<br>No	1<br>2 Don't know

	**Check Box**	
At least one Yes in F1 to F5	☐	*Go to F6*
All No or don't know in F1 to F5	☐	*Go to F7*

Respondent identification Number ☐☐☐☐

F 6	Coronary artery disease, Stroke, Diabetes and Hypertension					
	First degree relative	Age of Occurence CAD	Age of Occurence Stroke	Whether diabetic Yes 1 No. 2	Whether hypertensive Yes 1 No. 2	Whether hypercholeste-rolemic Yes 1 No. 2
F 6 a	☐☐	☐☐	☐☐	☐	☐	☐
F 6 b	☐☐	☐☐	☐☐	☐	☐	☐
F 6 c	☐☐	☐☐	☐☐	☐	☐	☐
F 6 d	☐☐	☐☐	☐☐	☐	☐	☐
F 6 e	☐☐	☐☐	☐☐	☐	☐	☐
F 6 f	☐☐	☐☐	☐☐	☐	☐	☐
F 6 g	☐☐	☐☐	☐☐	☐	☐	☐
F 6 h	☐☐	☐☐	☐☐	☐	☐	☐

Code 01 Mother 02 Father
11 Brother 1, 2 2 Brother 2, 13 Brother 3 and so on,
21 Sister 1, 22 Sister 2, 23 Sister 3 and so on,
31 Son 1, 32 Son 2 and 33 Son 3 and so on
41 Daughter 1, 42 Daughter 2, 43 Daughter 3 and so on
Code 99 if don't know

F 7	Number of siblings of the participant (excluding the participant)				
F 7 a	Number alive	☐☐			
F 7 b	Number deceased	☐☐			
F 7 c	Total	☐☐			

Respondent identification Number | | | | |

**Treatment History (Section T)** ചികിത്സാ ചരിത്രം (സെക്ഷൻ ടി)		
**T 1**	Have you ever been told to have high blood pressure?<br>നിങ്ങൾക്ക് ഉയർന്ന രക്തസമ്മർദ്ദമുള്ളതായി എപ്പോഴെങ്കിലും പറഞ്ഞുകേട്ടിട്ടുണ്ടോ?	Yes 1<br>No 2 → *Skip to T5*
**T 2**	If yes, for how long have you been known to have high blood pressure?<br>അതെ എന്നാണെങ്കിൽ, എത്രകാലമായി നിങ്ങൾക്ക് ഉയർന്ന രക്തസമ്മർദ്ദമുള്ളതായി അറിയാം?	Weeks 1 ☐☐<br>Months 2 ☐☐<br>Years 3 ☐☐
**T 3**	For the last one year was there any occasion when your blood pressure was recorded high in medical tests?<br>കഴിഞ്ഞ ഒരു കൊല്ലമായി വൈദ്യപരിശോധനയിൽ നിങ്ങളുടെ രക്തസമ്മർദ്ദം ഉയർന്നതായി രേഖപ്പെടുത്തപ്പെട്ട വല്ല സന്ദർഭവും ഉണ്ടായിരുന്നോ?	Yes 1<br>No 2
**T 4**	Are you currently receiving any treatment for high blood pressure?<br>ഉയർന്ന രക്തസമ്മർദ്ദത്തിന് ഇപ്പോൾ എന്തെങ്കിലും ചികിത്സ ലഭിച്ചുകൊണ്ടിരിക്കുന്നുണ്ടോ?	Yes 1<br>No 2
**T 5**	When was your blood pressure last measured by a health professional / Doctor<br>ആരോഗ്യ തൊഴിൽ വിദഗ്ദർ / ഡോക്ടർ നിങ്ങളുടെ രക്തസമ്മർദ്ദം അവസാനമായി പരിശോധിച്ചത് എന്നായിരുന്നു?	Within past 12 months 1<br>1-5 years ago 2<br>Not within past 5 yrs. 3
**T 6**	Were you ever found to have diabetes in a medical test?<br>വൈദ്യപരിശോധനയിൽ എപ്പോഴെങ്കിലും നിങ്ങൾക്ക് പ്രമേഹമുള്ളതായി കണ്ടെത്തിയിട്ടുണ്ടോ ?	Yes 1<br>No 2 → *Skip to T 9*
**T 7**	If yes, how long have you been known to have diabetes?<br>അതെ എന്നാണെങ്കിൽ എത്രകാലമായി പ്രമേഹമുള്ളതായി നിങ്ങൾക്ക് അറിയാം?	Weeks 1 ☐☐<br>Months 2 ☐☐<br>Years 3 ☐☐
**T 8**	Are you currently receiving any treatment for diabetes?<br>ഇപ്പോൾ പ്രമേഹത്തിന് എന്തെങ്കിലും ചികിത്സ ലഭിക്കുന്നുണ്ടോ?	Yes 1<br>No 2
**T 9**	Have you had your blood sugar measured in the last one year ?<br>കഴിഞ്ഞ ഒരു കൊല്ലത്തിൽ രക്തത്തിലെ പഞ്ചസാരയുടെ അളവ് നിങ്ങൾ പരിശോധിച്ചിരുന്നുവോ?	Yes<br>No
**T 10**	Have you ever been advised treatment for heart disease ?<br>നിങ്ങൾക്ക് ഹൃദ്രോഗത്തിന് എപ്പോഴെങ്കിലും ചികിത്സ നിർദ്ദേശിക്കപ്പെട്ടിട്ടുണ്ടോ?	Yes 1<br>No 2 → *Skip to T 12*
**T 11**	Are you currently receiving any treatment for heart disease ?<br>ഹൃദ്രോഗത്തിന് ഇപ്പോൾ എന്തെങ്കിലും ചികിത്സ നിങ്ങൾക്കു ലഭിച്ചുകൊണ്ടിരിക്കുന്നുണ്ടോ?	Yes 1<br>No 2

Respondent identification Number ☐☐☐☐

**T 12**	Have you ever been told to have high Cholesterol ?<br>നിങ്ങൾക്ക് ഉയർന്ന കൊളസ്ട്രോൾ ഉള്ളതായി എപ്പോഴെങ്കിലും പറയപ്പെട്ടിട്ടുണ്ടോ?	Yes 1<br>No 2 → *Skip to T 16*
**T 13**	Are you currently receiving any treatment for lipid abnormalities?<br>രക്തത്തിലെ കൊഴുപ്പിന്റെ ആധിക്യത്തിന് നിങ്ങൾക്കിപ്പോൾ എന്തെങ്കിലും ചികിത്സ ലഭിച്ചുകൊണ്ടിരിക്കുന്നുണ്ടോ?	Yes 1<br>No 2
**T 14**	How long have you been diagnosed to have lipid abnormalities?<br>നിങ്ങൾക്ക് രക്തത്തിലെ കൊഴുപ്പിന്റെ ആധിക്യം ഉള്ളതായി രോഗനിർണ്ണയം ചെയ്യപ്പെട്ടിട്ട് എത്രകാലമായി?	Weeks 1 ☐☐<br>Months 2 ☐☐<br>Years 3 ☐☐
**T 15**	Are you on treatment with statin group of drugs?<br>സ്റ്റാറ്റിൻ വിഭാഗത്തിൽപ്പെട്ട ഔഷധങ്ങളുടെ ചികിത്സയിലാണോ നിങ്ങൾ?	Yes 1<br>No 2
**T 16**	When was your blood cholesterol last measured ?<br>നിങ്ങളുടെ രക്തത്തിലെ കൊളസ്ട്രോൾ അവസാനമായി പരിശോധിച്ചത് എപ്പോഴാണ്?	Within past 12 months 1<br>1-5 years ago 2<br>Not within past 5 yrs. 3

**Awareness Questions (Section W)** ബോധവത്ക്കരണ ചോദ്യങ്ങൾ (സെക്രഷൻ ഡബ്ല്യൂ)				
**W 1**	Do you think smoking causes<br>പുകവലി ഇനിപ്പറയുന്നവയ്ക്ക് കാരണമാകുമെന്ന് നിങ്ങൾ കരുതുന്നുണ്ടോ?<br><br>(Multiple response)<br>Record all responses	Heart attacks<br>Strokes<br>Cancers<br>Don't know	A<br>B<br>C<br>D	
**W 2**	Do you think high blood pressure causes<br>ഉയർന്ന രക്തസമ്മർദ്ദം ഇനിപ്പറയുന്നവയ്ക്ക് കാരണമാകുമെന്ന് നിങ്ങൾ കരുതുന്നുണ്ടോ?<br><br>(Multiple response)<br>Record all responses	Heart attacks<br>Strokes<br>Cancers<br>Don't know	A<br>B<br>C<br>D	
**W 3**	Do you think one has high blood pressure if<br>ഇനിപ്പറയുന്നവ ഉണ്ടെങ്കിൽ ഒരാൾക്ക് ഉയർന്ന രക്തസമ്മർദ്ദമുണ്ടെന്നു നിങ്ങൾ കരുതുന്നുണ്ടോ?	Systolic BP $\geq$ 140 and/or diastolic BP $\geq$ 90<br>Systolic BP $\geq$ 160 and/or diastolic BP $\geq$ 95<br>Systolic BP $\geq$ 180 and/or diastolic BP $\geq$ 100<br>Don't know	1<br>2<br>3<br>9	
**W 4**	Do you think cut off for normal BP in an adult depends on age ?<br>മുതിർന്നയാളിൽ സാധാരണ നിലയിലുള്ള ബി. പി. യ്ക്കു വേണ്ടിയുള്ള കട്ടോഫ് പ്രായത്തെ ആശ്രയിച്ചിരിക്കുന്നുവെന്നു നിങ്ങൾ കരുതുന്നുണ്ടോ?	Yes<br>No	1<br>2	

Respondent identification Number □□□□

W 5	What is the most important dietary intervention to control high blood pressure<br>ഉയർന്ന രക്തസമ്മർദ്ദം നിയന്ത്രിക്കാനുള്ള ഏറ്റവും പ്രധാനപ്പെട്ട ആഹാരരീതിയുടെ ഇടപെടൽ എന്താണ്?	Avoid coconut oil<br>Eat vegetarian diet<br>Eat fruits and vegetables<br>Restrict salt<br>Don't know	1<br>2<br>3<br>4<br>9	
W 6	Do you think diabetes causes<br>പ്രമേഹം ഇനിപ്പറയുന്നവ ഉണ്ടാക്കുമെന്ന് നിങ്ങൾ കരുതുന്നുണ്ടോ?<br>(Multiple response)<br>Record all responses	Heart attacks<br>Kidney disease<br>Strokes<br>Cancers<br>Don't know	A<br>B<br>C<br>D<br>E	
W 7	Desirable level of total cholesterol in a person without heart disease is<br>ഹൃദ്രോഗമില്ലാത്ത ഒരു വ്യക്തിയിലെ ടോട്ടൽ കൊളസ്ട്രോളിന്റെ അഭിലഷണീയമായ അളവ് ഇനിപ്പറയുന്നതാണ്.	Less than 200 mg/Dl<br>Less than 230 mg/Dl<br>Less than 250 mg/Dl<br>Don't know	1<br>2<br>3<br>9	
Code 9 if don't know				

	Rose Questionnaire (Section Q)<br>റോസ് ചോദ്യാവലി (സെക്ഷൻ ക്യൂ)	Sub Section A : Effort Pain<br>ഉപവിഭാഗം എ : അദ്ധ്വാനം മൂലമുള്ള വേദന	
Q 1	Have you ever had any pain or discomfort in your chest?<br>നെഞ്ചിൽ നിങ്ങൾക്ക് എപ്പോഴെങ്കിലും വേദനയോ അസ്വസ്ഥതയോ ഉണ്ടായിട്ടുണ്ടോ?	Yes<br>No	1 → *Skip to Q 3*<br>2
Q 2	If No, have you ever had any pressure or heaviness in your chest<br>ഇല്ല എന്നാണെങ്കിൽ നെഞ്ചിൽ എപ്പോഴെങ്കിലും മർദ്ദമോ ഭാരമോ ഉണ്ടായിരുന്നിട്ടുണ്ടോ?	Yes<br>No	1<br>2 → *Skip to Q 15*
Q 3	Do you get it when you<br>ഇനിപ്പറയുന്നതു ചെയ്യുമ്പോൾ നിങ്ങൾക്കത് ഉണ്ടാകാറുണ്ടോ?	Walk uphill കുന്നിൻമുകളിലേക്കു നടക്കുക<br>Walk hurry ധൃതിയിൽ നടക്കുക<br>Both രണ്ടും<br>Never ഒട്ടുമില്ല	1<br>2<br>3<br>4 → *Skip to Q 9*
Q 4	Do you get it when you walk at an ordinary pace on the level ?<br>സമതല ഭൂമിയിൽ സാധാരണ ചുവടുവെപ്പു വേഗത്തിൽ നടക്കുമ്പോൾ നിങ്ങൾക്കത് ഉണ്ടാകാറുണ്ടോ?	Yes<br>No	1<br>2 → *Skip to Q 9*
Q 5	What do you do if you get it while you are walking ?<br>നടക്കുമ്പോൾ അതുണ്ടാകുകയാണെങ്കിൽ നിങ്ങളെന്താണു ചെയ്യുക?	Stop<br>Slow down<br>Carry on	1<br>2<br>3 → *Skip to Q 9*
Q 6	If you stand still what happens to it<br>നിങ്ങൾ നിശ്ചലമായി നില്ക്കുമ്പോൾ അതിനെന്തു സംഭവിക്കുന്നു?	Relieved<br>Not relieved	1<br>0 → *Skip to Q 9*

Respondent identification Number ☐☐☐☐

**Q 7**	How soon ?<br>എത്രവേഗത്തിൽ?	10 mts or less<br>More than 10 mts	1<br>0 → *Skip to Q 9*
**Q 8**	Will you show me where it was ?<br>അതെവിടെയായിരുന്നുവെന്ന് നിങ്ങളെന്നെ കാണിക്കുമോ?<br><br>(Multiple response)<br>Record all responses<br>*Specify (others 1, 2, 3,)*<br>*GO to Q 15*	Stemum upper or middle<br>Stemum lower<br>Left anterior chest<br>Left arm<br>Other 1<br>Other 2<br>Other 3	A<br>B<br>C<br>D<br>E<br>F<br>G
	**Rose Questionnaire (Section Q)**	**Sub Section A (Optional)**	
**Q 9**	Have you had this pain or discomfort more than three times ?<br>ഈ വേദനയോ അസ്വസ്ഥതയോ നിങ്ങൾക്ക് മൂന്നിൽ കൂടുതൽ തവണ ഉണ്ടായിരുന്നുവോ?	Yes<br>No	1<br>2
**Q 10**	Does any other kind of exertion bring it on ? (Specify)<br>മറ്റേതെങ്കിലും തരത്തിലുള്ള അദ്ധ്വാനം അതുണ്ടാക്കുന്നുണ്ടോ? (ചൂണ്ടിക്കാണിക്കുക)	Yes<br>No	1<br>2
**Q 11**	Do any of these things tend to bring it on ?<br>ഇനിപ്പറയുന്ന ഏതെങ്കിലും കാര്യങ്ങൾ അത് വരുത്താറുണ്ടോ ?<br><br>(Multiple response)<br>Record all responses	Excitement or Emotion ക്ഷോഭമോ വികാരമോ A<br>Stooping കുനിയൽ B<br>Eating തിന്നൽ C<br>Breathing ശ്വസനം D<br>Cold wind തണുത്ത കാറ്റ് E<br>Coughing ചുമ F	
**Q 12**	Is it worse if you have a chest cold or bad cough ?<br>നെഞ്ചിൽ കഫക്കെട്ടോ കടുത്ത ചുമയോ ഉള്ളപ്പോൾ അത് കൂടുതലാണോ ?	Yes<br>No	1<br>2
**Q 13**	Would you describe it as a pain or discomfort<br>നിങ്ങളതിനെ വേദനയെന്നാണോ അസ്വസ്ഥതയെന്നാണോ വിവരിക്കുക?	Yes<br>No	1<br>2
**Q 14**	Would any of these words describe the sensation<br>ഈ സംവേദനത്തെ ഇനിപ്പറയുന്നവയിൽ ഏതെങ്കിലും വാക്കുകൾ വിവരിക്കുമോ?<br><br>(Multiple response)<br>Record all responses	Heaviness ഭാരം A<br>Burning പുകച്ചിൽ B<br>Tightness മുറുക്കം C<br>Stabbing കുത്തൽ D<br>Pressure മർദ്ദം E	

Respondent identification Number ☐☐☐☐

Q 15	Have you ever had a severe pain across the front of your chest lasting for half an hour or more ?<br>നെഞ്ചിന്റെ മുൻഭാഗത്ത് വിലങ്ങനെ അര മണിക്കൂറോ അതിൽ കൂടുതലോ നീണ്ടുനിൽക്കുന്ന വേദന നിങ്ങൾക്ക് എപ്പോഴെങ്കിലും ഉണ്ടായിട്ടുണ്ടോ?	Yes<br>No	1<br>2
Q 16	How many of these attacks have you had<br>അവ എത്രയെണ്ണം നിങ്ങൾക്കുണ്ടായിട്ടുണ്ട്?	Yes<br>No	1<br>2

Q16a		Date	Duration	Other information
	First attack			
	Latest attack			

Q 17	Have you ever had an electrical recording of your heart ?<br>നിങ്ങളുടെ ഇ. സി. ജി. എപ്പോഴെങ്കിലും രേഖപ്പെടുത്തിയിട്ടുണ്ടോ ?	Yes<br>No	1<br>2
Q 18	Did you see a doctor because of this pain ?<br>ഈ വേദന മൂലം നിങ്ങൾ ഡോക്ടറെ കണ്ടിരുന്നുവോ?	Yes<br>No	1<br>2

To be filled by the principal investigator

Q 19	Effort Pain : If yes to Q 08 or Q 09<br>or Q 10 and Q 11	Yes<br>No	1<br>0	☐
Q 20	If no to Q 4<br>Ir yes to Q 4	Yes<br>No	1<br>0	☐
Q 21	Possible Infraction : If yes to Q 29	Yes<br>No	1<br>0	☐

Hostory of Hospital admission for ACS or Undergoing procedures for CAD (Section H)

H 1	Have you ever been hospitalized for Acute Coronary Syndrome (ACS) ?<br>അക്യൂട്ട് കൊറോണറി സിൻഡ്രോം എന്ന രോഗനിർണ്ണയത്തോടെ നിങ്ങൾ എപ്പോഴെങ്കിലും ആശുപത്രിയിൽ പ്രവേശിക്കപ്പെട്ടിട്ടുണ്ടോ ?	Yes<br>No<br>Don't know	1<br>0<br>2	*Ask to produce documents*<br>→ *Skip to T 1*
H 2	When was the first episode of hospitalization for ACS ?<br>എ സി എസു മായി ആശുപത്രിയിൽ പ്രവേശിപ്പിക്കപ്പെട്ട ആദ്യസംഭവം എപ്പോഴായിരുന്നു?	Within past 1 year<br>1 to 5 years ago<br>More than 5 years ago	1<br>0<br>2	
H 3	Have you ever undergone coronary angiography ?<br>നിങ്ങൾ എപ്പോഴെങ്കിലും കൊറോണറി ആൻജിയോഗ്രാഫിക്കു വിധേയമായിട്ടുണ്ടോ?	Yes<br>No<br>Don't know	1<br>0<br>2	*Ask to produce documents*
H 4	Have you ever undergone 64 slice CT angiography ?<br>64 സ്ലൈസ് സി ടി ആൻജിയോഗ്രാഫിക്ക് എപ്പോഴെങ്കിലും നിങ്ങൾ വിധേയമായിട്ടുണ്ടോ?	Yes<br>No<br>Don't know	1<br>0<br>2	*Ask to produce documents*
H 5	Have you ever undergone exercise stress electrocardiogram in the past ?<br>കഴിഞ്ഞ കാലങ്ങളിൽ എപ്പോഴെങ്കിലും നിങ്ങൾ വ്യായാമ സമ്മർദ്ദ ഇലക്ട്രോ കാർഡിയോ ഗ്രാമിനു വിധേയമായിട്ടുണ്ടോ?	Yes<br>No<br>Don't know	1<br>0<br>2	*Ask to produce documents*
H 6	Have you ever undergone ECHO ?<br>നിങ്ങൾ എപ്പോഴെങ്കിലും ECHO നു വിധേയമായിട്ടുണ്ടോ	Yes<br>No<br>Don't know	1<br>0<br>2	*Ask to produce documents*

Respondent identification Number ☐☐☐☐

Treatment Procedures (Section T)			
T 1	Have you ever been subjected to special procedures for treatment of heart disease ?	Yes<br>No	1<br>2 → *Skip to M 1*
T 1 a	PTCA ?	Yes<br>No	1 *Ask to produce documents*<br>2
T 2 b	CABG?	Yes<br>No	1 *Ask to produce documents*<br>2
T 3 c	Thrombolysis ?	Yes<br>No	1 *Ask to produce documents*<br>2

Physical Measurements

Height and weight			Coding Column
M 1	Technician ID Code		☐☐☐
M 2	Height	(in Centimetres)	☐☐☐.☐
M 3	Weight<br>if too large for scale, code 666.6	(in Kilograms)	☐☐☐.☐
**Waist**			
M 4	Technician ID		☐☐☐
M 5	(For women) Are you pregnant?	Yes 1<br>No 2	→ *Skip to M 7*
M 6	Waist circumference	(in Centimetres)	☐☐☐.☐

Blood pressure			Coding Column
M 7	Technician ID		☐☐☐
M 8	Device ID for blood pressure ?		☐☐☐
M 9	Cuff size used	Small<br>Normal<br>Large	1<br>2<br>3
M 10a	Reading 1 **Systolic BP**	Systolic mmHg	☐☐☐
M 10b	**Diastolic BP**	Diastolic mmHg	☐☐☐
M 11a	Reading 2 **Systolic BP**	Systolic mmHg	☐☐☐
M 11b	**Diastolic BP**	Diastolic mmHg	☐☐☐
M 12a	Reading 3 **Systolic BP**	Systolic mmHg	☐☐☐
M 12b	**Diastolic BP**	Diastolic mmHg	☐☐☐
M 13	**Pulse rate (per minute)**	Last reading	☐☐☐

Respondent identification Number ☐☐☐☐

**Biochemical Parameters (Section B)**		
**B 1**	Fasting Blood Sugar (mg / dL)	☐☐☐
**B 2**	Total Cholesterol (mg / dL)	☐☐☐
**B 3**	Triglyceride (mg / dL)	☐☐☐
**B 4**	HDL Cholesterol (mg / dL)	☐☐☐

Name of investigator.. Code ☐☐☐

Signature of investigator with date

Name of editor.. Code ☐☐☐

Signature of editor with date

Name of supervisory staff.. Code ☐☐☐

Signature of supervisory staff with date

CSI Kerala CRP Study Questionnaire

Name of Participant :

Age : 		Sex : 		Participant ID. No.

Household ID. No. : 		Date :

ECG	
Physical Measurements	
Questionnaire	
Biochemical Parameters	
Consent Form	
ABI	
Special Lab	

No CAD	
Probable CAD	
Definite CAD	
