# Supplementary material for: Prevalence of metabolic syndrome and its risk factors in Kerala, South India: Analysis of a community based cross-sectional study
Source: PLoS One. 2018 Mar 27;13(3):e0192372. doi: 10.1371/journal.pone.0192372 (PMC5870937; doi:10.1371/journal.pone.0192372)
Supplement: S2 Table — Prevalence estimates have been standardized for age and adjusted for sex and urban-rural distribution (% and 95% confidence interval). (PDF) [file pone.0192372.s002.pdf]

	Rural	Urban	p-value	Women	Men	p-value	Total
**Physical Activity**							
**Engagement in household chores**	**74.5**	**68.5**	<0.001*	**94.5**	**45.7**	<0.001*	**71.6**
	72.3-76.7	65.6-71.4		93.1-95.8	41.8-49.6		69.1-74.2
**Engagement in leisure time physical activity** (minutes per week)							
None	**72.0**	**58.1**		**80.2**	**48.5**		**65.3**
	69.4-74.6	54.2-62.1		77.6-82.9	44.5-52.4		62.1-68.6
1- 149	**9.5**	**14.6**		**8.9**	**15.4**		**12.0**
	7.5-11.5	11.5-17.7		6.8-10.9	12.4-18.5		9.4-14.5
>150	**18.5**	**27.3**	<0.001*	**10.9**	**36.1**	<0.001*	**22.7**
	16.2-20.8	23.9-30.7		9.0-12.8	32.2-39.9		19.9-25.5
**Activity preference** (engagement > once per week)							
Outdoor games	**6.7**	**6.0**	<0.001*	**0.8**	**12.7**	<0.001*	**6.4**
	5.0-8.3	4.1-7.9		0.2-1.3	9.6-15.9		4.6-8.1
Cycling	**5.0**	**2.8**	<0.001*	0.4	**7.9**	<0.001*	**4.0**
	3.7-6.4	1.8-4.1		0.0-1.2	5.9-10.0		2.8-5.3
Treadmill	**0.4**	**0.9**	0.024*	**0.5**	**0.8**	0.176	**0.6**
	0.1-0.8	0.0-1.8		0.0-1.2	0.0-1.5		0.0-1.3
Walking	**18.0**	**32.8**	<0.001*	**17.4**	**33.9**	<0.001*	**25.1**
	15.6-20.5	29.1-36.6		14.9-19.9	30.2-37.5		22.1-28.2
Active outdoor work	**3.9**	**2.3**	<0.001*	**1.5**	**5.0**	<0.001*	**3.2**
	2.7-5.2	1.1-3.5		0.9-2.2	3.1-6.8		1.9-4.4
**Diet**							
**Dietary preference**							
Non-vegetarian	**96.4**	**93.7**		**94.6**	**95.7**		**95.1**
	95.3-97.5	91.9-95.5		93.1-96.0	94.2-97.2		93.6-96.6
Vegetarian	**3.6**	**6.3**	<0.001*	**5.4**	**4.3**	0.075	**4.9**
	2.5-4.7	4.5-8.1		4.0-6.9	2.8-5.8		3.4-6.4
**Fruits and vegetables** (servings per day)							
5 or more	**2.3**	**4.6**		**3.2**	**3.6**		**3.4**
	1.3-3.4	2.8-6.3		1.9-4.5	2.2-5.1		2.0-4.8
2 to 4	**55.4**	**70.6**		**63.0**	**62.4**		**62.7**
	52.2-58.6	66.7-74.5		59.7-66.2	58.5-66.3		59.1-66.3
0 to 1	**42.2**	**24.7**	<0.001*	**33.8**	**33.9**	0.669	**33.8**
	39.0-45.4	21.0-28.5		30.6-37.0	30.1-37.7		30.4-37.3
**Fish**							
Fish consumption at least once per week	**95.7**	**92.3**	<0.001*	**93.5**	94.6	0.105	**94.1**
	94.5-96.9	90.2-94.3		92.0-95.0	92.9-96.3		92.5-95.6
**Oil** (% participants using each type)							
Coconut oil	**93.7**	**92.7**	0.159	**92.6**	**94.0**	0.060	**93.2**
	92.1-95.4	90.5-95.0		90.6-94.6	92.1-95.8		91.3-95.2
Palm oil	**16.3**	**26.5**	<0.001*	**22.0**	20.2	0.125	**21.2**
	14.0-18.7	22.9-30.1		19.2-24.9	17.2-23.3		18.2-24.2
Sunflower oil	**6.1**	**16.5**	<0.001*	**11.1**	**11.0**	<0.001*	**11.1**
	4.5-7.8	13.4-19.6		8.9-13.4	8.6-13.5		8.7-13.4
Other oils (refined vegetable, gingelly, or olive)	**0.4**	**1.0**	0.009*	**0.8**	**0.5**	0.096	**0.7**
	0.0-0.8	0.4-1.5		0.3-1.4	0.1-0.8		0.2-1.1
**Salt** (intake > once per week)							
Adding salt to rice	**33.5**	**27.6**	<0.001*	**30.8**	**30.5**	0.848	**30.7**
	30.4-36.6	23.7-31.5		27.6-34.0	26.8-34.3		27.2-34.1
Pickle consumption	**62.9**	**60.9**	0.146	**60.9**	**63.2**	0.091	**62.0**
	59.9-65.9	57.0-64.8		57.8-64.0	59.4-67.0		58.5-65.4
Papad consumption	**67.3**	**62.1**	<0.001*	**62.0**	**68.0**	<0.001*	**64.8**
	64.3-70.2	58.1-66.0		58.8-65.2	64.3-71.6		61.4-68.2
Salted fish consumption	**41.0**	**27.0**	<0.001*	**36.5**	**31.8**	<0.001*	**34.3**
	37.8-44.2	23.1-30.9		33.1-39.8	28.0-35.6		30.7-37.8
